# An extension of the localist representation theory: grandmother cells are also widely used in the brain

**DOI:** 10.3389/fpsyg.2013.00300

**Published:** 2013-05-24

**Authors:** Asim Roy

**Affiliations:** Department of Information Systems, Arizona State UniversityTempe, AZ, USA

## On grandmother cells

Based on considerable neurophysiological evidence, Roy (2012) proposed the theory that localist representation is widely used in the brain, starting from the lowest levels of processing. Grandmother cells are a special case of localist representation. In this article, I present the theory that grandmother cells are also widely used in the brain. To support the proposed theory, I present neurophysiological evidence and an analysis of the concept of grandmother cells. Konorski (1967) first predicted the existence of grandmother cells (he called them “gnostic” neurons)—single neurons that respond to complex stimuli such as faces, hands, expressions, objects, and so on. The term “grandmother cell” was introduced by Jerry Lettvin in 1969 (Barlow, 1995).

The notion of grandmother cells is very controversial in neuro and cognitive sciences. Barlow (2009, p. 320) claims that grandmother cells exist and “can now be recorded from and studied reliably.” Bowers (2009) has also claimed that the brain uses grandmother cells to code for objects and concepts. However, Plaut and McClelland (2010) and Quian Quiroga et al. (2008; Quian Quiroga et al., 2010) have vigorously opposed the notion of grandmother cells in the brain.

### The concept of grandmother cells

Grandmother cells have been characterized in a variety of ways. Here I reference some:

- Gross (2002, p. 512): “*The term “grandmother cell” refers to a neuron that would respond only to a specific, complex, and meaningful stimulus, that is, to a single percept or even a single concept. As originally conceived, a grandmother cell was multimodal, but the term came to be used mostly for representing a visual percept*.”
- Barlow (2009, p. 309): “*The term grandmother cell started as a fanciful name for a high-level neuron that might enable us to experience complex perceptions and discriminate among them. The concept included invariance of response for changes in some variables as well as selectivity of response for others, together with the idea that these cells are created by processing at a hierarchy of levels*.”
- Bowers (2009, p. 223): “*In sum, the key claim of localist coding schemes is that a given unit (neuron) codes for one familiar thing (and does not directly contribute to the representation of anything else), and that it is possible to interpret the output of a single unit in a neural network*.”

By localist representation, Bowers implies grandmother cells.

### The grandmother cell represents a complex, abstract concept

For this article, let's start with the stricter definition that a grandmother cell represents a specific and complex concept, not merely a percept, in a multimodal invariant way. Thus, the basic grandmother cell notion is about encoding a complex concept within a single cell in an invariant way. And abstract categories—such as animals, cars, and houses—are, without question, complex concepts. Category-type concepts, therefore, are part of (or included in) the notion of grandmother cells. In other words, the grandmother cell notion is not necessarily restricted to just object-related concepts (such as the concepts of *Jennifer Aniston*, *Saddam Hussein*, and *Eiffel Tower*), but is inclusive of the whole range of complex concepts. In a more general sense, grandmother cells are fundamentally about abstraction and generalization. We, therefore, need to find evidence for the brain's ability to generalize and encode an abstract idea in a single cell in an invariant way. For object-related concepts, there is now plenty of evidence for multimodal invariant abstract cells that encode such concepts (such as the concepts of *Oprah Winfrey*, *Saddam Hussein*, and *Sydney Opera*) and the evidence is presented in section The Evidence for Modality Invariant Object-related Concept Cells. There is also plenty of evidence for category-related concept cells, although they are primarily for visual stimuli. But, in any modality, category cells represent an abstraction about a set of objects or features and reflect brain's ability to generalize, which is the essential feature of grandmother cells. And these category cells are invariant to shape, size, and other features of the objects in that category. Thus, finding a category cell in any modality can be counted as evidence for grandmother cells. Section The Evidence for Category Cells presents evidence for category cells in the brain.

In the context of localist theory (Roy, 2012), grandmother cells are a special type of localist cells. Roy (2012) has already shown that localist cells in the brain have “meaning and interpretation.” Therefore, the output of a grandmother cell is also interpretable and there is no need to revisit the interpretability issue here.

## The evidence for modality invariant object-related concept cells

In some experiments, reported in Quian Quiroga et al. (2009) and others, they found that single medial temporal lobe (MTL) neurons can encode an object-related concept irrespective of how it is presented—visual, textual, or sound. They check the modality invariance properties of a neuron by showing the subjects three different pictures of the particular individual or object that a unit responds to and their spoken and written names. In these experiments, they found (Quian Quiroga et al., 2009, p. 1308) “*a neuron in the left anterior hippocampus that fired selectively to three pictures of the television host Oprah Winfrey and to her written (stimulus 56) and spoken (stimulus 73) name …. To a lesser degree, the neuron also fired to the actress Whoopi Goldberg*. *None of the other responses were significant, including other text and sound presentations*.” They also found a neuron in the entorhinal cortex of a subject that responded (Quian Quiroga et al., 2009, p. 1308) “*selectively to pictures of Saddam Hussein as well as to the text “Saddam Hussein” and his name pronounced by the computer …. There were no responses to other pictures, texts, or sounds*.”

Quian Quiroga (2012, p. 588) reports: “Another neuron responded to Halle Berry—*even when she was masked as Catwoman*, a character she played in one of her movies …. These and many other examples suggest that MTL neurons encode an *abstract representation* of the concept triggered by the stimulus. This claim was tested more conclusively by presenting the written names of these persons or objects to the subjects, and it was found that *a large proportion of MTL neurons did indeed respond to both the pictures and the written names of a particular individual* (*or object*). For example, the hippocampal neuron that fired selectively to pictures of Halle Berry responded also to the letter string “HALLE BERRY” (*and not to other names*). Moreover, *the selective responses of these neurons could be triggered by stimuli in other sensory modalities*, such as the name of a person pronounced by a synthesized voice ….”

Suthana and Fried (2012, p. 428) report similar findings: “*Thus, a neuron may respond to a picture of the Sydney Opera House and exhibit no response to 50 other landmarks, yet also respond to many permutations and physically different representations of the Sydney Opera House, seen in color, in black and white, or from different angles. In fact, the neuron may also respond to the iconic representation, namely the words “Sydney Opera,” which is obviously different in its visual properties compared with the image of this landmark. Recently, it was shown that this invariance crosses modalities, meaning that MTL neurons may exhibit a selective and “invariant” response to a particular stimulus out of 100 images and do so independently of the sensory modality (visual image, audio, or written iconic representations) through which the stimulus was presented ….*”

Quian Quiroga et al. (2008) estimate that 40% of MTL cells are tuned to such explicit representation.

### The evidence for modality invariant concept cells based on “thinking” about a concept

In the experiment by Cerf et al. (2010), epilepsy patients played a game to control the display of two superimposed images through four MTL neurons. Before the experiment, researchers identified four MTL neurons in each patient that responded selectively to four different images. One of the four images was randomly selected to become the target image. Each trial started with a short display of the target image (say of Jennifer Aniston) followed by an overlaid hybrid image of the target and one of the other three images (a distractor image, say of James Brolin). The patient was then told to enhance the target image by focusing his/her “thoughts” on it. (Note: Perhaps internal imagery in the brain was used by patients when asked to “think” about a target image.) The initial visibility of both images was at 50% and the visibility of an image was increased or decreased every 100 ms based on the firing rates of the four MTL neurons. In general, if the firing rate of one neuron was higher compared to the other, the image associated with that neuron became more visible. The trial was terminated when either one of the two images was fully visible or after a fixed time limit. The subjects successfully reached the target, which means the target image was fully visible, in 596 out of 864 trials (69.0%; 202 failures and 66 timeouts).

Note that if the target image was of Jennifer Aniston that means that they found a neuron that responded to Jennifer Aniston images and not to others. And that same Jennifer Aniston neuron was activated by the patient by simply “thinking” about Jennifer Aniston. That indicates the multimodal invariance property of that Jennifer Aniston cell—multimodal because it is triggered by both visual and internal stimuli. And this experiment was widely replicated—it was performed many times on many patients.

## The evidence for category cells

Cells that represent categories have been found in both humans and animals. These cells reflect brain's ability to generalize and create abstractions. The invariance property is reflected in the fact that these cells respond to a class of objects—objects with varying shapes, sizes, and other features. Fried et al. (1997) found some MTL neurons that respond selectively to gender and facial expression and Kreiman et al. (2000) found MTL neurons that respond to pictures of particular categories of objects, such as animals, faces, and houses. Recordings of single-neuron activity in the monkey visual temporal cortex led to the discovery of neurons that respond selectively to certain categories of stimuli such as faces or objects (Logothetis and Sheinberg, 1996; Tanaka, 1996; Freedman and Miller, 2008).

I quote Freedman and Miller (2008): “*These studies have revealed that the activity of single neurons, particularly those in the prefrontal and posterior parietal cortices (PPCs), can encode the category membership, or meaning, of visual stimuli that the monkeys had learned to group into arbitrary categories.*”

Lin et al. (2007) report finding “nest cells” in mouse hippocampus that fire selectively when the mouse observes a nest or a bed, regardless of the location or environment.

Gothard et al. (2007) found single neurons in the amygdala of monkeys that responded selectively to images of monkey faces, human faces, and objects as they viewed them on a computer monitor. They found one neuron that responded in particular to threatening monkey faces. Their general observation is (p. 1674): “*These examples illustrate the remarkable selectivity of some neurons in the amygdala for broad categories of stimuli*.”

Thus the evidence is substantial that category cells exist in the brain and that the brain can abstract and generalize.

## Conclusion

Grandmother cells are about abstracting complex concepts and using single units to encode and represent them. There is obviously an efficiency aspect to this. First, they provide information in an abstracted, summarized, and tractable form that can be easily exploited by other units of the brain. Second, it avoids interpreting an underlying pattern over and over again by different parts of the brain, where the pattern could be distributed over hundreds of thousands of units at lower levels. Thus, simplification, concreteness, automation, and computational efficiency are the key advantages of grandmother cells or complex concept cells.

Another powerful feature of grandmother and complex concept cells is easy and efficient access to cognitive level information, information that is interpretable and has meaning at a higher level of thought. Cognitive level information is no longer elusive, but easily available through these complex concept cells. The physical embodiment of cognitive level information within a set of complex concept cells makes cognition and thought very real and easily tractable within the brain. That makes complex concept cells extremely valuable and fundamental to the processes that deal with cognition and thought.
